# Selective Electrooxidation of Glycerol Into Value-Added Chemicals: A Short Overview

**DOI:** 10.3389/fchem.2019.00100

**Published:** 2019-02-25

**Authors:** Christophe Coutanceau, Stève Baranton, Roméo S. Bitty Kouamé

**Affiliations:** IC2MP, MediaCat Group, UMR CNRS-Université de Poitiers n°7285, Poitiers, France

**Keywords:** activity, catalysts, electrooxidation, glycerol, HPLC, *in situ* IR, selectivity

## Abstract

A comprehensive overview of the catalysts developed for the electrooxidation of glycerol with the aim of producing selectively value-added compounds is proposed in the present contribution. By presenting the main results reported in the literature on glycerol electrooxidation in acidic and alkaline media, using different kinds of catalytic materials (monometallic catalysts based on platinum group metals and non-noble metals, multimetallic alloys, or modification of surfaces by adatoms, etc.) and under different experimental conditions, some general trends concerning the effects of catalyst composition and structure, of reaction medium and of the electrode potential to enhance the activity for the glycerol oxidation reaction and of the selectivity toward a unique value-added product will be presented and discussed. The objective is to provide a guideline for the development of electrochemical systems which allow performing the electrooxidation of glycerol at the rate and selectivity as high as possible.

## Introduction

Glycerol is now considered as a largely available, inexpensive, and inherently renewable compound, which could be used as platform molecule for fine chemistry. The trans-esterification reaction in presence of methanol (methanolysis) of vegetable oil to produce biodiesel leads to ca. 10 wt.% of glycerol as side product (Clacens et al., 2002; Dasari et al., 2005). In the case of bio-ethanol production by anaerobic fermentation, significant amount of glycerol is also formed (several wt.%) (Aldiguier et al., 2004). The increasing demand of biofuels worldwide inevitably generated large production of glycerol in amounts much higher than that needed for industries, and therefore important stocks (Ciriminna et al., 2014). It can then be considered as a waste from biofuel industries. For this reason, several heterogeneous, homogeneous, or bio- catalysis processes have been developed to transform glycerol into energy and/or value-added compounds (Behr et al., 2008; Ilie et al., 2011) such as esters, glycerol carbonates, ethers, acetals, or ketals (Pagliaro, 2017). These new usages of glycerol provide a good opportunity for cutting down the production costs of biofuels, which are currently more expensive than fossil fuels and need state exemption to taxation to reach the market.

Amongst the species derived from glycerol, all C3 oxidation compounds (glyceraldehyde, dihydroxyacetone, glyceric acid, tartronic acid, hydroxypyruvic acid, and mesoxalic acid) have economic and/or industrial interests (Behr et al., 2008). But these compounds are generally produced either by enzymatic (microbial) processes (in the case of dihydroxyacetone da Silva et al., 2009, as an example) or by using strong oxidants (permanganate, nitric acid, chromic acid, etc.). Microbial processes lead generally to high selectivity, but to low conversion rates; moreover, other important drawbacks of such processes concern microbe separation, control of byproducts and disposal of waste water produced by the industrial process (Aarthy et al., 2014). The use of stoichiometric oxidants does not allow the control of the reaction selectivity and leads to the formation of large number of products and by-products (Behr et al., 2008). For these reasons, it has been proposed to develop catalytic processes to increase the conversion and simultaneously to control the selectivity of the oxidation reactions (Carrettin et al., 2004). Generally, the catalytic reactions are performed at ca. 50–60°C pressurized with oxygen (Katryniok et al., 2011; Villa et al., 2015), which can be detrimental for the selectivity.

**Figure 1:**
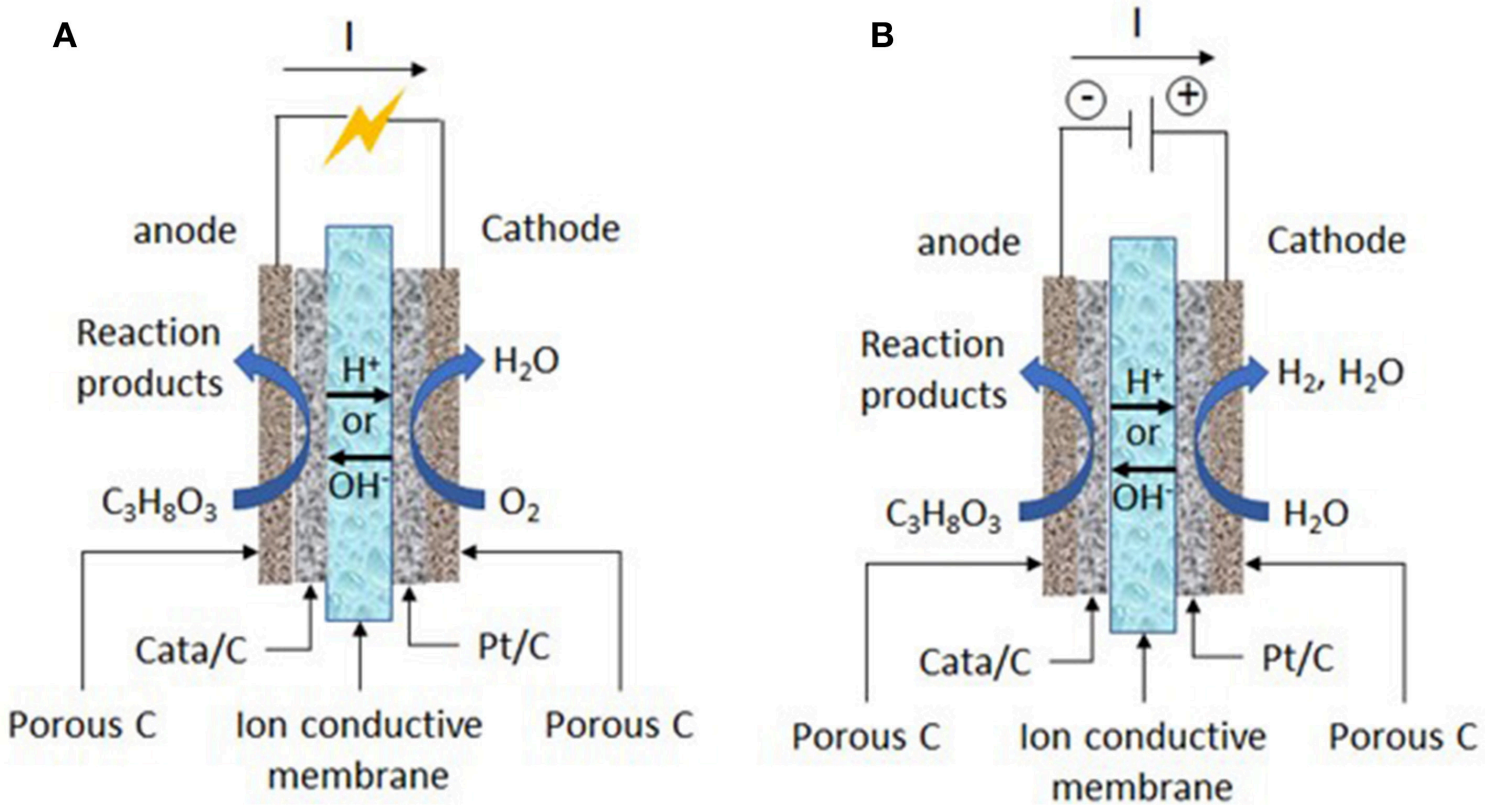
Working principles of **(A)** an acidic or alkaline solid polymer glycerol fuel cell and **(B)** an acidic or alkaline solid polymer glycerol electrolysis cell.

In this context, electrocatalysis and electrochemical methods for glycerol oxidation have certainly a role to play. In electrochemical methods, the glycerol oxidation can be performed at room temperature, i.e., without external power supply for the heater and without temperature control system, and in the only presence of water and electrolyte (Na^+^ or K^+^/OH^−^ in alkaline media and H^+^/${\text{SO}}_{4}^{2-}$ or ${\text{ClO}}_{4}^{-}$ in acidic media). Co-reactants are water as oxygen source, which avoid to work under pressurized oxygen conditions or in presence of oxidative agents in the reaction medium (in electrochemical methods, water molecules can be activated at the solid electrode materials to provide extra-oxygen atoms for the oxidation reaction of organic compounds), solid electrode materials (immobilized catalysts), and electrons, which can make electrocatalysis a more sustainable process than catalysis. Moreover, the activity of electrocatalysts and selectivity of the reaction toward a given reaction product can be accurately tuned and enhanced through the control of the structure/composition of the electrocatalyst and of the electrode potential. Indeed, these parameters control both the glycerol and water adsorption (activation) at the catalytic surface and further the route of oxidation and the reaction product distribution (Simões et al., 2012; Coutanceau et al., 2014; Zalineeva et al., 2014; Cobos-Gonzalez et al., 2016). At last, the electrochemical oxidation of glycerol into value-added compounds at the anode of an electrosynthesis reactor (Figure 1) can be accompanied by electric energy production (in fuel cell mode) (Bambagioni et al., 2009; Benipal et al., 2017) or hydrogen production (in electrolysis cell mode) (Bambagioni et al., 2010; Chen et al., 2014), which both can bring higher economical interest to the process.

The current contribution aims at presenting a short overview of the achievements in glycerol electrooxidation toward value-added compounds. By presenting the main results reported in acidic and alkaline media, using different kinds of catalytic materials and under different experimental conditions, some trends concerning the selective electrooxidation of glycerol will be presented. The objective is to provide a guideline for the development of electrochemical systems allowing performing the electrooxidation of glycerol at the highest rate and highest selectivity possible. This requirement is mandatory for decreasing the E-factor, as determined by Sheldon (Sheldon, 2017), of the electrochemical processes down to 0 by only providing valuable outputs (value-added chemicals and energy or hydrogen) from an industrial waste.

## Experimental

### Synthesis of Catalysts by a Water-Oil-Microemulsion Method (Boutonnet et al., 1982; Coutanceau et al., 2008)

Hexachloroplatinic acid hexahydrate (H_2_PtCl_6_•6H_2_O), tetrachloroauric acid trihydrate (HAuCl_4_•3H_2_O), potassium tertrachloropalladate (K_2_PdCl_4_), nickel chloride (NiCl_2_) and bismuth chloride (BiCl_3_) purchased form from Alfa Aesar (99 % purity) were used as metal salt precursors for the syntheses. Aqueous solutions were prepared in ultrapure water (MilliQ, Millipore, 18.2 MΩ cm) with total concentration in metal salts of 0.1 mol L^−1^ from appropriate weights of metal salts to reach the desired atomic ratios. A 1.6 mL aliquot of a metal salt solution was placed into a flask containing a homogenous mixture of 37.0 g of n-heptane (Sigma Aldrich, HPLC grade) as oil phase and 16.1 g of polyethylene glycol dodecyl ether (Brij® L4, Sigma Aldrich) as surfactant, under continuous stirring conditions until a stable and translucent microemulsion was obtained. The microemulsion consisted in nanodroplets of aqueous solution containing the metal salts protected by the surfactant and in suspension in the continuous n-heptane phase. The metal salts in nanodroplets were then reduced by addition to the mixture of 100 to 200 mg of solid sodium borohydride (NaBH_4_, 99 % purity, Sigma Aldrich) to reach a large excess. After the reaction was completed (hydrogen evolution has stopped), appropriate amount of carbon powder (Vulcan XC72, CABOT) pre-treated under N_2_(g) at 400 °C for 4 h was directly added to the colloidal solution to reach a metal loading of ca. 40 wt.%. After sonication for ca. 15 min, the mixture was filtered under vacuum on a hydrophilic polyvinylidene difluoride (PVDF) membrane (0.22 mm Durapore membrane filter from Millipore). The carbon-supported metal nanoparticle material was washed several times with acetone and ultrapure water and dried overnight in an oven at 60°C. At last, the catalytic powder was thermally treated for 2 h at 200°C under air atmosphere to remove any remaining surfactant.

### Electrochemical Measurements

The electrochemical setup consisted in a computer-controlled Voltalab PGZ 402 potentiostat. The solutions were prepared from NaOH (Semiconductor grade 99.99% purity, Sigma-Aldrich), ultrapure water and glycerol (Sigma-Aldrich, Reagent Plus, purity ≥ 99%). The electrochemical experiments were carried out at 20°C in N_2_-purged (U-quality, l'Air liquid) 0.1 or 1.0 mol L^−1^ NaOH electrolyte containing 0.1 mol L^−1^ glycerol, using a conventional thermostated three-electrode electrochemical cell. The working electrode was prepared by deposition of a catalytic ink onto a glassy carbon disc (0.071 cm^−2^ geometric surface area). The catalytic ink consisted in the dispersion of 17.7 mg of catalytic powder in 2.117 mL of ultrapure water, 0.529 mL of 2-propanol (LC-MS CHROMASOLV, Fulka), and 0.354 mL of Nafion solution (5 wt % Nafion perfluorinated resin solution in aliphatic alcohols, Sigma Aldrich). After homogenization by sonication (about 30 s), 3 μL of catalytic ink was dipped using a microsyringe on the freshly polished glassy carbon disc, leading to a metal loading of 100 μg_metal_ cm^−2^. The solvent was then evaporated in a stream of pure nitrogen at room temperature. The counter electrode was a glassy carbon plate (4 cm^2^ geometric surface area), and the reference electrode was a reversible hydrogen electrode (RHE).

### *In situ* Infrared Spectroscopy

*In situ* Fourier transform infrared spectroscopy (FTIRS) experiments were performed on a Bruker IFS 66 FTIR spectrometer modified for beam reflection on the electrode surface at a 65° incident angle. To remove interferences from atmospheric water and CO_2_ the beam path was evacuated. An Infrared Associates liquid nitrogen-cooled HgCdTe detector was used. The spectral resolution was 4 cm^−1^, and each spectrum was obtained by averaging 512 spectra recorded for 35 s. Spectra were recorded every 0.05 V during the linear voltammetry carried out at 1 mV s^−1^ from 0.1 to 1.2 V vs. RHE. The experimental details of the electrochemical setup for SPAIRS (single potential alteration IR spectroscopy) are described elsewhere (Beden and Lamy, 1988; Kabbabi et al., 1998). The method of spectrum normalization involved that negative absorption bands corresponded to the formation of species at the electrode surface and positive absorption bands corresponded to the consumption of species at the electrode surface.

### Chronoamperometry Experiment

The electrolysis test was carried out in a recirculation mode at 20°C with a 2.0 M glycerol and 0.5 M NaOH aqueous solution in a 5 cm^2^ geometric surface area filter press-like single electrolysis cell at a flow rate of 20 mL min^−1^. The *U*_cell_(*t*) curves were recorded by using a DC power supply (E3614A from Agilent) to fix the cell voltage and Digital Multimeters (34405A from Agilent) to record the cell voltage and the applied current. The electrodes (cathode: 1.6 mg cm^−2^ Pt loading; anode: 1.6 mg cm^−2^ metal loading; both electrodes: 20 wt.% PTFE, 0.8 mg cm^−2^ Nafion) for chronoamperometry measurements were prepared by depositing an ink consisting in a mixture of Nafion (5 wt. % from Aldrich) solution, ultrapure water and the catalytic powder, on a carbon porous layer (CPL). The CPLs (4 mg cm^−2^ of a mixture of carbon powder and 20 wt. % PTFE) were made from a carbon cloth (Electrochem Inc.) on which was brushed an ink made of Vulcan XC 72 carbon powder and PTFE dissolved in isopropanol. The electrodes (cathode and anodes) were separated by a simple blotting paper and mechanically pressed in the electrolysis cell.

## Electro-Oxidation of Glycerol in Acidic Media

### Mono-Metallic Catalytic Materials

Because very few electrode materials are sufficiently stable or active in acidic medium, the electro-oxidation of glycerol has essentially been studied on platinum and platinum-based surfaces. It has been considered for a long time that platinum was unavoidable in such media to activate the dissociative adsorption of alcohols at its surface (Hogarth and Ralph, 2002; Venancio et al., 2002; Léger et al., 2005), leading to adsorbed species which could be further oxidized and desorbed at relatively high rates. However, it has been proposed recently that gold could also present some activity toward glycerol electrooxidation in acidic medium (Valter et al., 2018). This result was very surprising as previous works established that gold wasn't an active material in acidic media for alcohol oxidation (Beden et al., 1987; Kwon et al., 2011a). This discrepancy was attributed by Valter et al. to the nature of the electrolyte used for the oxidation reaction (Valter et al., 2018). Beden et al. (1987) and Kwon et al. (2011b) performed their experiments in 0.5 M H_2_SO_4_, whereas Valter et al. (2018) used 0.1 M HClO_4_ as supporting electrolyte. These last authors proposed that the stronger adsorption of sulfate ions than that of perchlorate ions on gold surface (Angerstein-Kozlowska et al., 1986) could be responsible for the lack of Au surface activity for glycerol electrooxidation in 0.5 M H_2_SO_4_ electrolyte. Based only on a DFT study, they explored the most thermodynamically favorable pathway on Au (111) in the potential range below 1.0 V vs. RHE, where no O and/or OH species are adsorbed on the gold surface. They proposed the formation of dihydroxyacetone and 2,3-dihydroxy-2-propenal from the low electrode potential value of 0.39 V vs. RHE (reversible hydrogen electrode), the formation of CO from 0.5 V vs. RHE and glyceraldehyde from 0.6 V vs. RHE, both these last species being expected to remain adsorbed on the surface. But, no experimental evidences were given to support this reaction pathway. Moreover, the presence of CO indicated that the breaking of the C-C bond occurred, meaning low selectivity in C3 products, and moreover the activity, as determined from the current densities recorded by cyclic voltammetry for 0.1 M glycerol electrooxidation in 0.1 M HClO_4_ electrolyte, remained very low (which could be explained by the blocking of the surface by CO and glyceraldehyde for potential lower than 1.0 V vs. RHE).

Up to now, platinum still remains the best catalytic materials in acidic media either for the oxygen reduction reaction (Gasteiger et al., 2005) in a fuel cell, or for the water reduction reaction (Mamaca et al., 2012) in an electrolysis cell and for alcohol oxidation reactions (Léger et al., 2005) in both cases. From kinetics study based on chromatographic analysis of reaction products formed at different potentials in 0.5 M H_2_SO_4_ on a bare Pt electrode, Roquet et al. proposed several mechanisms of glycerol oxidation according to the potential range (Roquet et al., 1994). At low potentials, where the platinum surface is not or weakly covered by hydroxide species, Pt led to very high selectivity toward glyceraldehyde (ca. 97 % at 0.75 V vs. RHE), whereas at potential higher than 1.0 V vs. RHE, where Pt is covered by an oxide layer, the selectivity decreased (56 % at 1.30 V vs. RHE) and other products such as glyceric acid, formic acid, and glycolic acid were detected. The proposed mechanism involved the O-adsorption as first adsorption step:

no OH coverage:

(1) $$\begin{array}{l} \text{Pt}+\;{\text{C}}_{2}{\text{H}}_{5}{\text{O}}_{2}-{\text{CH}}_{2}\text{OH}\;\to \;{\text{C}}_{2}{\text{H}}_{5}{\text{O}}_{2}-{\text{CH}}_{2}\text{O}-\text{Pt}\\ \;+\;1\;{\text{e}}^{-}\;+\;1\;{\text{H}}^{+} \end{array}$$

(2) $$\begin{array}{l} {\text{C}}_{2}{\text{H}}_{5}{\text{O}}_{2}-{\text{CH}}_{2}\text{O}-\text{Pt}\;\to \;\text{Pt}\;+\;{\text{C}}_{2}{\text{H}}_{5}{\text{O}}_{2}-\text{CHO}\\ \;+\;1\;{\text{e}}^{-}\;+\;1\;{\text{H}}^{+} \end{array}$$

weak OH coverage:

(3) $$\begin{array}{r} \text{Pt }+\;{\text{H}}_{2}O\;\to \;Pt-OH\;+\;H^{+}\;+\;e^{-} \end{array}$$

(4) $$\begin{array}{l} {\text{C}}_{2}{\text{H}}_{5}{\text{O}}_{2}-{\text{CH}}_{2}\text{O}-\text{Pt}\;+\;\text{Pt}-\text{OH}\;\to \;{\text{C}}_{2}{\text{H}}_{5}{\text{O}}_{2}-\text{COOH}\\ \;+\;2\text{Pt}\;+\;2{\text{H}}^{+}\;+\;2{\text{e}}^{-} \end{array}$$

O coverage:

(5) $$\begin{array}{r} 2\text{Pt }+\;2{\text{H}}_{2}O\;\to \;2\;PtO\;+\;4H^{+}\;+\;4e^{-} \end{array}$$

(6) $$\begin{array}{l} 2\text{ PtO + }{\text{C}}_{2}{\text{H}}_{5}{\text{O}}_{2}-{\text{CH}}_{2}\text{OH}\\ \;\to \;{\text{CH}}_{2}\text{OH}-\text{COH}-\text{CHOH + 2 }{\text{H}}^{+}\text{ + }2{\text{e}}^{-}\\ \;|\;|\\ \text{ Pt}-\text{O O}-\text{Pt} \end{array}$$

(7) $$\begin{array}{l} {\text{CH}}_{2}\text{OH}-\text{COH}-\text{CHOH }\to \text{ 2Pt + }{\text{CH}}_{2}\text{OH}-\text{COOH}\\ \;|\;|\\ \text{ Pt}-\text{O O}-\text{Pt}\\ \text{ + HCOOH} \end{array}$$

Kwon et al. (2012) performed same kind of measurements on a carbon supported Pt/C catalyst and obtained also a very high selectivity toward glyceraldehyde and small amount of glyceric acid for potential lower than 0.9 V. For higher potentials, they observed also a decrease in glyceraldehyde selectivity, with the formation of compounds of higher oxidation levels such as glyceric acid, formic acid, and glycolic acid, both the latter involving the breaking of the C-C bond. They detected also by *in situ* FTIRS measurements the production of CO_2_ at potentials higher than 1.1 V vs. RHE. According to their study the increase of the selectivity toward glycolic and formic acids was accompanied with a drastic decrease of the selectivity toward glyceraldehyde and glyceric acid; they proposed that glyceraldehyde and glyceric acid acted as reaction intermediates, conversely to the mechanism proposed by Roquet et al. (1994) were glycolic and formic acids appeared as primary products (Equation 7). More recently, high selectivity toward glyceraldehyde at 1.136 V vs. RHE (99.2) for the oxidation of 0.1M glycerol in 0.5 M H_2_SO_4_ have been reported on highly-dispersed Pt nanoclusters loaded on 3D graphene-like microporous carbon (Lee et al., 2019). Actually, these authors proposed that the glycerol/Pt molar ratio (195 or 433), the applied anode potential (1.097 V or 1.136 V) and the reaction time (10 or 6 h) could change the glycerol conversion from 91.8 to 2.3 % together with an increase in glyceraldehyde selectivity from 2.5 to 99.2 (Kim et al., 2014; Lee et al., 2019).

Both the mechanisms proposed by Roquet et al. (1994) and Kwon et al. (2012) involve the ability of platinum to break the C-C bond, at least at medium and high electrode potentials. However, Schnaidt et al. (2011) studied the adsorption/oxidation of glycerol, glyceraldehyde, and glyceric acid in 0.5 M H_2_SO_4_ using combined spectroelectrochemical DEMS/ATR-FTIRS (differential electrochemical mass spectroscopy/attenuated total reflectance—Fourier transform infrared spectroscopy) set-up. Highly sensitive *in situ* ATR-FTIR was used to monitor the development of glycerol adlayer as a function of potential or time, whereas online DEMS was used to detect the volatile products formed. First these authors observed for glycerol adsorption/oxidation ATR-FTIRS absorption bands assigned to the formation of linearly and multiply bonded CO_ads_, adsorbed glyceroyl and adsorbed glycerate species. The dissociative adsorption of glycerol into CO_ads_ occurred for potential between 0.2 and 0.6 V vs. RHE. For potentials higher than 0.5 V vs. RHE, the formation of CO_2_ could be detected although in small amount indicating that incomplete oxidation of glycerol into C3 compounds prevailed under these conditions. The formation of CO_ads_ at low potentials and of CO_2_ at potentials higher than 0.5 V vs. RHE was confirmed by Gomes et al. from investigation of the electrooxidation of 0.1 M glycerol in 0.1 M H_2_SO_4_ and 0.1 M HClO_4_ electrolyte on low Miller-index Pt crystalline surface orientations, Pt (100), Pt (110), and Pt (111), followed by *in situ* FTIR spectroscopy (Gomes et al., 2012). They also observed the formation of carbonyl species for potentials higher than 0.3 V vs. RHE. From their study on the adsorption/oxidation of glyceraldehyde and glyceric acid. Schnaidt et al. (2011) proposed also that glyceraldehyde was an intermediate for the formation of both CO_ads_ and glyceric acid. They found also that glyceric acid was a dead end in the electrochemical oxidation of glycerol. But, the detection of adsorbed CO species from glycerol adsorption speaks for a mechanism starting with a C-adsorption step rather than an O-adsorption step as proposed by Roquet et al. (1994). Moreover, the proposition that glyceric acid is a dead end in the electrochemical oxidation of glycerol is in contradiction with the mechanism proposed by Kwon et al. (2012) for the formation of glycolic, oxalic, formic acid and CO_2_ at high potentials.

The study of glycerol electrooxidation as a function of its concentration on platinum surfaces in 0.1 M HClO_4_ performed by Gomes et al. (2013) shed light on some mechanistic aspects of this reaction. First, they confirmed that the adsorption of glycerol led to the formation of CO_ads_ from low potentials. Second, they showed that the electrooxidation of glycerol on Pt was strongly influenced by the concentration of this alcohol. At high glycerol concentrations, a denser coverage than at low concentrations of the Pt surface by glycerol adsorbates occurred, which led to delay the formation of adsorbed OH species on the platinum surface and further the CO_ads_ desorption into CO_2_ through a Langmuir-Hinshelwood mechanism (CO_ads_ + OH_ads_ → CO_2_ + H^+^ + e^−^), and glyceraldehyde, which is the preferentially formed products at low and medium potentials, contributed significantly to the formation of the CO_ads_ layer, in agreement with the observation of Schnaidt et al. (2011). Moreover, Gomes et al. (2013) found that the formation of carboxylic acids at medium and high potentials occurred in a parallel pathway and was not affected by the glycerol concentration. At high potentials carboxylic acids were partly oxidize and participated to the production of CO_2_.

(Kongjao et al., 2011) performed electrochemical reforming of a 0.5 M glycerol at a current of 4.5 A in an electrochemical reactor fitted with two Pt electrodes (66.49 and 124.34 cm^2^ geometric surface area for the anode and cathode, respectively) in a monochamber cell without separation of the cathodic and anodic compartments. Therefore, their analysis by gas chromatography indicated the formation of reduction products (at the cathode) and oxidation products (at the anode) from glycerol. Concerning the oxidation products, glyceraldehyde, C1 and C2 acids were obtained as reaction products. No C3 carboxylic acid was detected. It is likely that the anode potential (which was not controlled in the experiments) reached for a current of 4.5 A was very high (higher than 1.0 V vs. RHE). Under such conditions, the C-C bond occurred in a large extent leading to low weight carboxylic acids and to CO_2_.

**Figure 2:**
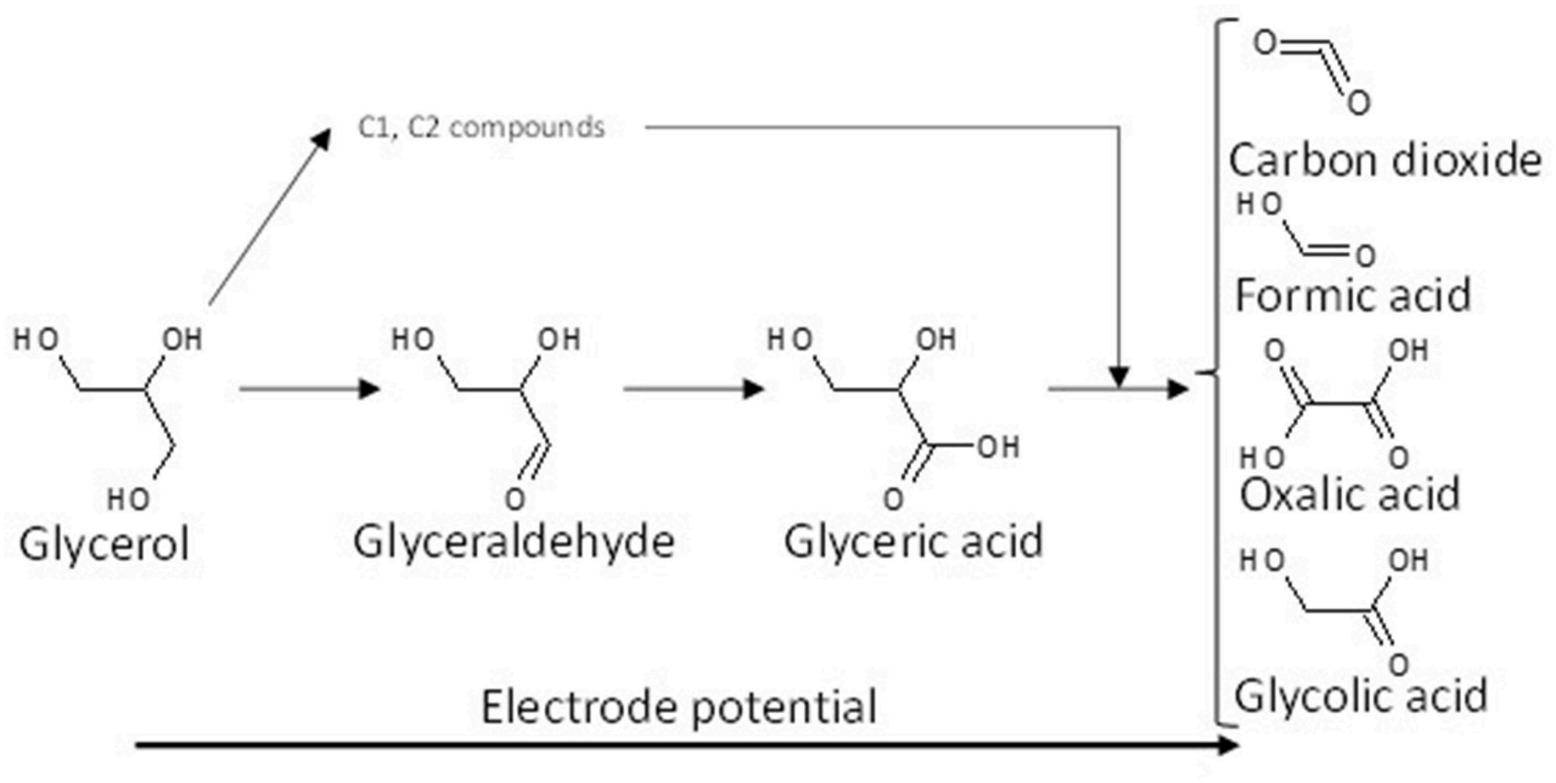
Scheme of the reaction pathways for the electrooxidation of glycerol on platinum in acidic media.

Although it is obvious that further investigations are needed to determine the mechanism of glycerol electro-oxidation on platinum surface, a general reaction scheme on platinum can be proposed (Figure 2).

### Multi-Metallic Catalytic Materials

Studies on glycerol adsorption and electro-oxidation at platinum surfaces have revealed that the type and the density of adsorbed species from glycerol on the catalytic surface greatly influenced the mechanism and the formation of final products. A classical approach to increase the activity and the selectivity of platinum consists in modifying its surface by other atoms, alloyed or deposited as ad-atoms. It is indeed known for a long time that the modification of Pt surfaces by foreign atoms has an important effect on the amount and composition of chemisorbed species. This effect will change the course of electrooxidation of adsorbed species and change the activity and selectivity of the catalyst (Podlovchenko et al., 1966; Smirnova et al., 1988). On platinum surfaces, it has been evidenced that adsorbed species, such as CO or aldehydes, were formed from low electrode potentials and blocked the surface. It has also been evidenced that the removal of such adsorbed species needed the presence of adsorbed OH species to allow further oxidation through the Langmuir-Hinshelwood mechanism. Therefore, by changing the balance between the sites blocked by adsorbed species (Pt sites) and the sites activating water to form adsorbed OH species (foreign atom sites), the activity and the selectivity of the catalyst can be enhanced by allowing a bi-functional mechanism occurring (Watanabe and Motoo, 1975). This effect is called the third body effect (Schmidt et al., 2000). Another effect of the presence of foreign atoms at the platinum surface consists in diluting the Pt surface atoms, which limits the mean number of adjacent Pt atoms and further leads to change the nature of adsorbed species and the strength of their adsorption (Adzic, 1984).

Ruthenium is a co-catalyst often used for the electro-oxidation of alcohols. In particular, PtRu/C catalysts displayed higher activity and stability than Pt/C catalyst for the glycerol electro-oxidation (Kim et al., 2011). The best atomic composition in term of activity was found to be Pt_5_Ru_5_/C, with an onset potential for glycerol oxidation of 0.448 V vs. RHE against 0.635 V vs. RHE for Pt/C. Chronoamperometry measurements at 1.1 V vs. RHE were performed for 7 h for the oxidation of 0.1 M glycerol in 0.5 M H_2_SO_4_ on Pt/C and Pt_5_Ru_5_/C (1.0 mg cm^−2^ metal loading) as anodes at 60 °C (Kim et al., 2017). Higher current densities were obtained with the Pt_0.5_Ru_0.5_/C anode than with the Pt/C anode, confirming the higher activity of the former catalyst. Moreover, the product distributions determined using high-performance liquid chromatography were different for both catalysts; glyceraldehyde, glyceric acid, and glycolic acid were detected for both catalysts but with different rates, and dihydroxyacetone was only detected with the Pt_5_Ru_5_/C catalyst. The authors determined the total mass balance and found that it was 100 % with the Pt/C catalyst, whereas it decreased to 80 % with the Pt_5_Ru_5_/C catalyst. They concluded that the Pt_5_Ru_5_/C catalyst also promoted the breaking of the C-C bond leading to the formation of formic acid and CO_2_.

**Figure 3:**
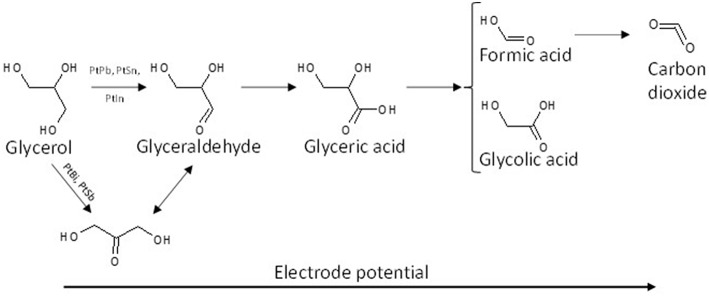
Scheme of the reaction pathways for the electrooxidation of glycerol on platinum modified by elements from the p-group in acidic media.

The effect of the modification of platinum surfaces by metals from p-group on the electro-oxidation of glycerol has also been studied. Bismuth is known for a long time to be an excellent co-catalyst for alcohol and biomass conversion by heterogeneous catalysis (Gallezot, 1997). Kwon et al. (2012) compared the activity and selectivity of a Pt/C, a Bi-modified Pt/C and a Pt/C catalyst in Bi-saturated solution. The onset potential for glycerol electrooxidation decreased from ca. 0.5 V to ca. 0.47 V and ca. 0.4 V vs. RHE for Pt/C, Bi-modified Pt/C and Pt/C in Bi-saturated solution, respectively. But the most important results were that the presence of bismuth in solution, and further the high Bi coverage of the Pt surface, avoided the formation of adsorbed CO species and oriented the reaction toward the activation of the secondary alcohol group to produce dihydroxyacetone with very high selectivity at low electrode potentials, whereas on pure Pt the formation of glyceraldehyde was predominant at low electrode potentials. The effect of the modification of platinum by adatoms irreversibly adsorbed on a carbon supported platinum electrode, antimony (Sb), lead (Pb), indium (In), and tin (Sn), has also been studied toward the glycerol electro-oxidation (Kwon et al., 2014). Sb as adatoms displayed the highest activity and allowed achieving a very high selectivity toward dihydroxyacetone from very low potentials, with an onset potential of ca. 0.35 V vs. RHE shifted by ca. 150 mV with respect to that with Pt/C). Both Sb and Bi promoted the secondary alcohol oxidation, whereas Pb, In, and Sn promoted the oxidation of the primary alcohol groups toward glyceraldehyde at low electrode potentials. For all catalysts, glyceric acid was detected at medium potentials and glycolic acid at high potentials. Figure 3 shows the general reaction scheme of glycerol electrooxidation on platinum modified by p-group elements. The study of Pb@Pt core-shell structures agreed with this reaction mechanism (Silva et al., 2016). The structural and geometric effects induced by the core-shell structure improved the direct oxidation of formic acid into carbon dioxide, limiting the CO surface coverage and hence increasing the catalytic activity. Although these studies are very interesting in a fundamental point of view, the implementation of such system for DHA production is likely avoided for cosmetic, food, or pharmaceutical applications because of the presence of antimony or bismuth in solution.

## Electro-Oxidation of Glycerol in Alkaline Media

In acidic media, the electro-oxidation of glycerol involves the use of platinum. The advantage of alkaline media is that kinetics of alcohol electrooxidation is faster than in acidic ones (Wang et al., 2003). Indeed, Koper et al. proposed that in alkaline media the first deprotonation step for the electrooxidation of alcohols to form alkoxide species was base catalyzed, and that the second deprotonation step depended on the catalytic ability of the surface (Kwon et al., 2011a). This could explain the higher activity toward alcohol electrooxidation not only of gold, but also of platinum in alkaline media than in acidic media. Then other metals than platinum can be used to perform this reaction. Moreover, non-noble metals are more stable in alkaline media. For example, gold, which displays no or very low activity in acidic media, becomes very active in alkaline media; nickel-based catalysts, which are not stable in acidic media, can be used in alkaline media. Hence, a larger panel of electrocatalytic materials, including platinum (Simões et al., 2011; Cobos-Gonzalez et al., 2016; Da Silva et al., 2017) palladium (Bambagioni et al., 2010; Simões et al., 2012; Wang et al., 2016), gold (Jeffery and Camara, 2010; Simões et al., 2010; Gomes et al., 2014; Ottoni et al., 2016), nickel (Oliveira et al., 2013, 2014; Lin et al., 2017; Houache et al., 2018), rhodium (Pagliaro et al., 2017), etc., -based catalysts, could be evaluated for the glycerol oxidation reactions.

### Non-platinum Group Metals

It is known that nickel is an active material for alcohol electrooxidation in alkaline media. It is generally admitted that the electrooxidation of alcohol starts simultaneously with the Ni^II^(OH)_2_ → Ni^III^OOH transition at high potentials. This transition occurs from the onset potential of ca. 1.3 V vs. RHE. Fleischmann et al. (1971, 1972) proposed that the early steps of alcohol electrooxidation in alkaline media on nickel occurred according to the following equations:

(8) $$\begin{array}{r} \text{Ni}{\left(\text{OH}\right)}_{2}\;+\;OH^{-}\;\to \;NiOOH\;+\;H_{2}O\;+\;e^{-} \end{array}$$

(9) $$\begin{array}{l} \text{NiOOH}\;+\;\text{R}-{\text{CH}}_{2}\text{OH}\;+\;{\text{OH}}^{-}\;\to \text{ Ni}{\left(\text{OH}\right)}_{2}\;+{\text{ H}}_{2}\text{O}\\ \;+\text{R}-\text{CHO}\;+{\text{e}}^{-} \end{array}$$

(10) $$\begin{array}{r} \text{R}-\text{CHO }+\;3\text{ O}{\text{H}}^{-}\;\to \;R-COO^{-}\;+\;2\;H_{2}O\;+\;2\;e^{-} \end{array}$$

Therefore, according to this mechanism the oxidation of glycerol could only occur for potentials higher than 1.3 V vs. RHE, i.e., in a very oxidative potential range. However, the onset potential of glycerol oxidation is shifted toward a lower potential of ca. 1.1 V vs. RHE, i.e. 200 mV less than the potential for the Ni^II^(OH)_2_ → Ni^III^OOH transition. This effect was already observed (Tehrani and Ab Ghani, 2012; Oliveira et al., 2013) and was attributed to the fact that the very early steps of the transformation of Ni(OH)_2_ to NiOOH was primordial to start the glycerol oxidation reaction. Houache et al. (2018) performed *in-situ* photo-elastic modulations infrared reflection absorption spectroscopy (PM-IRRAS) measurements coupled with electrochemical experiments for the oxidation at different constant potentials (from ca. 1.27 V to ca. 1.47 V vs. RHE, i.e., in the potential range of the early stages of glycerol oxidation) of 0.1 M glycerol in 1.0 M KOH aqueous electrolyte. They were able to analyze simultaneously the products formed on the Ni surface and in the bulk solution, and detected glyceraldehyde, carbonyl, carboxylate ions, and some carbon dioxide. They attributed the absorption band at ca. 1,700 cm^−1^ to glyceraldehyde and carbonyl species. However, according to Oliveira et al. (2013), the broad band at ca. 1,700 cm^−1^ is characteristic of the stretching vibration of the C=O carbonyl group in a C-COO^−^ structure. Therefore, in the potential range from 1.2 to 1.6 V vs. RHE, the electrooxidation of glycerol seems to involve the C-C bond breaking and leads mainly to the formation of formate and glycolate species, even if the formation of C3 carboxylates cannot be discarded. Indeed, Oliveira et al. (2013) confirmed these results by HPLC analysis of reaction products after long-term electrolysis at 1.6 V vs. RHE of 0.1 M glycerol in 0.1 M NaOH electrolyte. For higher potentials than 1.6 V vs. RHE, important production of CO_2_ was also observed by *in situ* infrared spectroscopy. The determination of reaction intermediates by *in situ* FTIRS and the product distribution by HPLC didn't point out the formation of glyceraldehyde, which was however stipulated by Fleischmann et al. (1971, 1972) as the second step of glycerol electro-oxidation (Equation 9). This could be due to the very oxidative anode potential (1.6 V vs. RHE for the long-term electrolysis), which made glyceraldehyde very reactive, oxidizing rapidly this molecule into glycerate, glycolate, formate, carbonate, and even CO_2_.

In order to increase both activity and selectivity, attempts were done to modify nickel by other metals, such as Co and Fe (Oliveira et al., 2013, 2014). The modification of Ni by Co led to the formation of larger amount of CO_2_, which was explained by the lower potential for the Co^II^(OH)_2_ → Co^III^OOH transition than for the Ni^II^(OH)_2_ → Ni^III^OOH transition. The modification of Ni by Co, Fe or CoFe did not change significantly the onset potential of glycerol electrooxidation which remained always higher than 1.1 V vs. RHE but had an effect on the reaction product distribution (Oliveira et al., 2014). But, the main products were still formate, glycolate and other carboxylates.

### Platinum Group Metals

Platinum, palladium and gold are the main studied materials from the platinum group metals for the electrooxidation of glycerol in alkaline media. The onset potential for glycerol electrooxidation is close to 0.4 V on both nanostructured Pt/C (Simões et al., 2010) and polycrystalline Pt disc (Kwon and Koper, 2010), whereas (Simões et al., 2010) showed that it was closed to 0.6 V vs. RHE at Pd/C and Au/C nanostructured catalysts. As in acidic media, platinum displays a better activity at lower electrode potentials. Consequently, it was measured that at low potential (from 0.4 to 0.8 V vs. RHE), the activation energy is lower on Pt than on Pd and Au (Habibi and Razmi, 2012). This trend reverts at high potential (> 0.8 V vs. RHE) and Au then possesses the lowest activation energy in addition to a good poisoning tolerance. *In situ* infrared spectroscopy measurements and HPLC analysis or reaction products were also performed on such materials.

**Figure 4:**
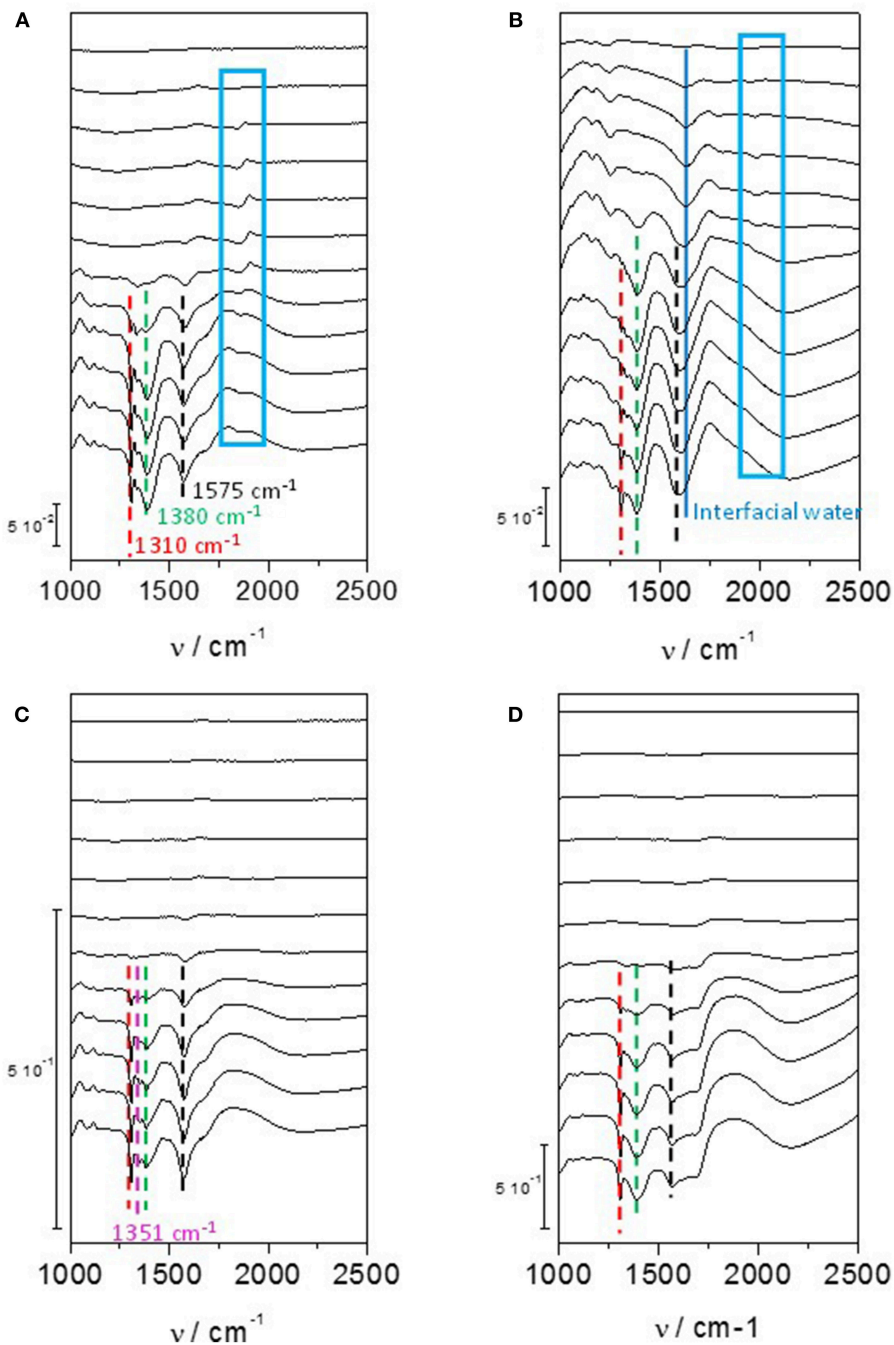
Infrared spectra recorded in the 1,200–2,500 cm^−1^ range for 0.1 M glycerol oxidation in 0.1 M NaOH from 0.05 V to 1.15 V vs. RHE on **(A)** Pd/C, **(B)** Pt/C, **(C)** Au/C, and **(D)** Pd_9_Bi_1_/C prepared by the Water-in-Oil microemulsion method (*T* = 20 °C, scan rate: 1 mV s^−1^, resolution 4 cm^−1^). The dotted bars show the infrared absorption band at ca. 1,310 cm^−1^ (red), ca. 1,380 cm^−1^ (green), ca. 1,175 cm^−1^ (black) and in the case of Au/C at ca. 1,351 cm^−1^ (pink). The dark blue plain line shows the position of the Infrared absorption band relative to interfacial water and the blue boxes shows the infrared absorption bands related to adsorbed CO species on Pd/C and Pt/C catalysts (Simões et al., 2010, 2011, 2012).

On a polycrystalline Pt disc electrode, Kwon and Koper (2010) showed that the first glycerol oxidation products was glycerate from 0.35 V to 0.8 V vs. RHE; then secondary products, glycolate and formate, were produced through glycerate oxidation and C-C bond breaking from 0.4 V vs. RHE; at last the ternary product, oxalate acid, coming from the oxidation of glycolate was detected from 0.6 V vs. RHE. In addition, tartronate formation coming from glycerate oxidation without C-C bond breaking was observed over the 0.6–1.0 V vs. RHE potential range. The same conclusion was obtained by (Simões et al., 2010, 2011, 2012) for glycerol electrooxidation on Pt/C nanostructured catalyst, but they observed also infrared absorption bands at ca. 1,150 cm^−1^ and 1,310 cm^−1^ that they attributed to the formation of glyceraldehyde (Figure 4A). These authors also showed that although the onset potential was shifted by ca. 200 mV toward higher potential, the same product as with platinum were obtained with Pd/C, i.e., mainly glyceraldehyde, glycerate with IR bands at ca. 1,380 cm^−1^ and 1,575 cm^−1^, tartronate and/or mesoxalate ions with IR band at ca. 1,575 cm^−1^ (Figure 4B). In addition, they observed the infrared absorption band at ca. 1,335 cm^−1^ relative to the formation of dihydroxyacetone with both catalysts, but with a very low relative intensity. It is worth to note that over the 0.05 to 1.15 V vs. RHE potential range of the experiments, no CO_2_ formation, giving rise to an infrared absorption band at ca. 2,343 cm^−1^ (Dailey et al., 1998; Dubau et al., 2003), was observed, whereas the presence of an absorption band at 1,950 cm^−1^ on Pt/C and 1,900 cm^−1^ on Pd/C catalyst, the positions and intensities of which are both dependent on the electrode potentials, was due to the formation of adsorbed CO species (bridge bonded CO) on platinum (Couto et al., 2001) and palladium (Jiang et al., 2001) surfaces, respectively. This means that these materials are able to break the C-C bond in a very low extent, to produce also C1 and C2 species which were not detected. All these *in situ* infrared results were confirmed by HPLC analysis at different potentials (Simões et al., 2011).

Simões et al. (2010, 2012), Kwon and Koper (2010), and Jeffery and Camara (2010) studied the electrooxidation of glycerol on Au surface. Combining HPLC with voltrammetry measurements at a polycrystalline Au electrode, Kwon and Koper (2010) detected first glycerate from 0.6 V vs. RHE and then glycolate and formate from 0.8 V vs. RHE. Using *in situ* infrared spectroscopy, (Simões et al., 2010, 2012) and Jeffery and Camara (2010) observed on nanostructured Au/C catalyst and polycrystalline Au electrode, respectively, an absorption band at ca. 1,351 cm^−1^ (Figure 4C) which corresponded to the formation hydroxypyruvate, in additions to that of glyceraldehyde and other carboxylates (Simões et al., 2010, 2012). The formation of hydroxypyruvate indicate that the secondary alcohol function is activated and oxidized into ketone on Au surfaces. At potentials higher than 1.3 V, the formation of CO_2_ occurred indicating the breaking of the C-C bonds and therefore the destruction of the carbon chain.

**Figure 5:**
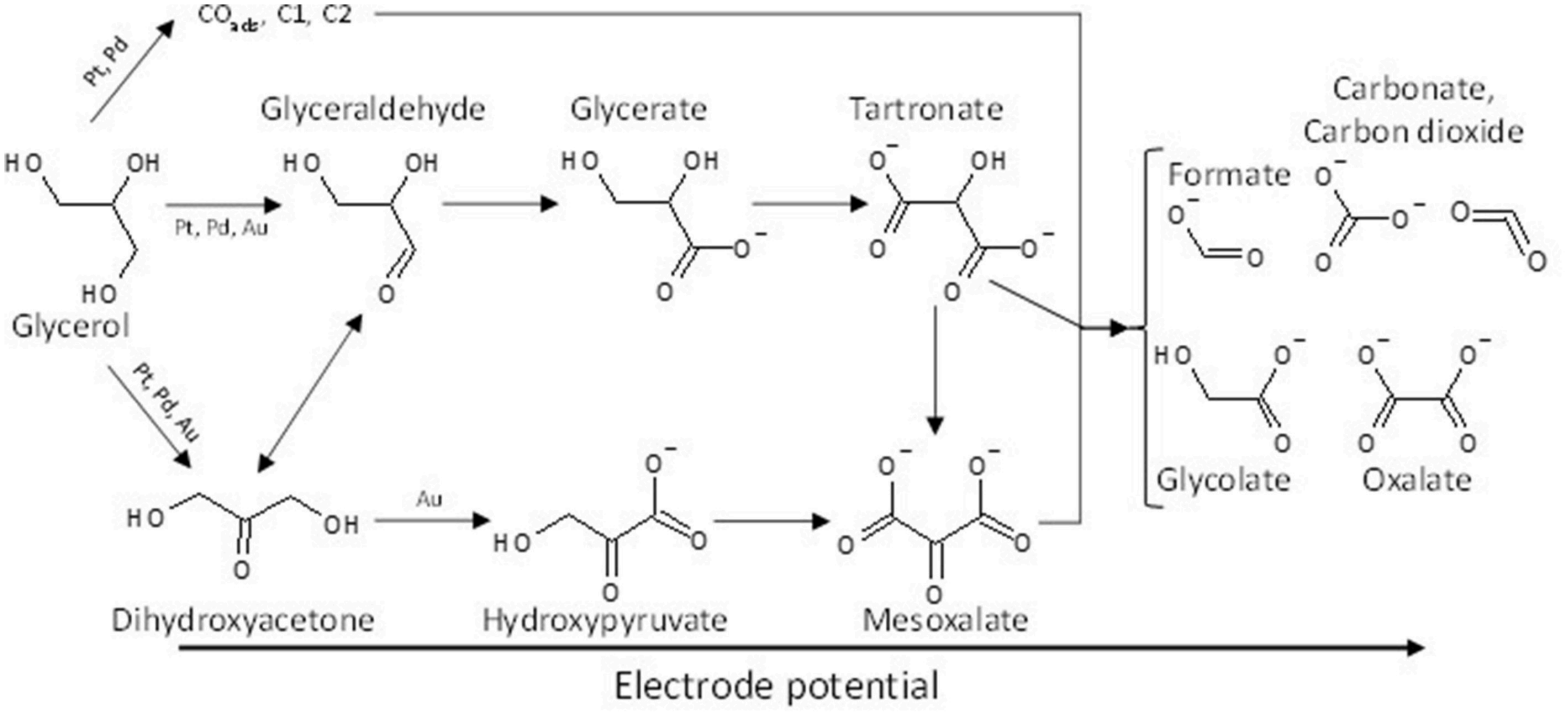
Scheme of the reaction pathways for the electrooxidation of glycerol on platinum, palladium and gold in alkaline media.

These observations indicate clearly that the nature of the catalytic metal can orient the reaction pathway. Platinum and palladium seem to favor the activation and oxidation of primary alcohol functions toward glyceraldehyde and further toward carboxylates, whereas on Au surfaces the secondary alcohol can be activated leading to the formation of hydroxypyruvate. Hydroxyacetone was also detected on Pt/C and Pd/C, but no hydroxypyruvate. Simões et al. (2012) proposed then that the isomerization equilibrium between dihydroxyacetone and glyceraldehyde could be displaced toward the formation of the aldehyde on the Pt/C and Pd/C catalysts comparatively to the Au/C catalyst. A general reaction scheme of glycerol electrooxidation on platinum group metals is proposed in Figure 5.

The use of platinum group metals as catalytic materials allowed to decrease the onset potential for glycerol electrooxidation and to increase the selectivity toward C3 oxidized compounds, compared to non-noble catalytic materials. This remark suggests that decreasing still more the onset potential for this reaction may help to increase the selectivity toward a given product. This can be made by varying the composition and the structure of Pt, Pd, and Au-based catalysts.

For example, Gomes et al. (2014) studied the effect of the modification of gold by silver. They found that the bimetallic catalysts led to lower onset potential for glycerol electrooxidation than the Au/C and to higher reaction rate. But, they also found that Ag addition influenced the mechanism of glycerol electrooxidation, favoring the C-C bond breaking, leading to the selective formation of formate.

The effect of the modification of Pd catalysts by metals from the d-group, such as Ni (Simões et al., 2010, 2012; Holade et al., 2013), Ru (Dash and Munichandraiah, 2015), Au (Simões et al., 2010, 2012; Ottoni et al., 2016), and Ag (Holade et al., 2013) has also been studied and the electrocatalytic activity and selectivity of bimetallic Pd_x_Au_x_/C, Pd_x_Ni_1−x_/C,Pd_x_Ru_1−x_/C and Pd_x_Ag_1−x_ catalysts toward glycerol electrooxidation have been estimated. Although it is admitted that the modification of Pd by Ni, Ru, Au, or Ag led to a shift of the onset of glycerol oxidation toward lower potentials than that on pure Au and Pd metals, i.e., an increase of the activity at lower electrode potentials, the selectivity of the catalysts was essentially the same as for pure Pd. Bambagioni et al. (2010) developed a very active Pd-(Ni-Zn)/C catalyst for glycerol electrooxidation and performed electrolysis measurements at room temperature for the oxidation of glycerol (10 wt%) in 2 M KOH at effective potentials between 0.6 and 0.7 V in an alkaline electrolyzer with a MEA made of a Pd–(Ni–Zn)/C/Ni mesh anode, an E-TEK Pt/C cathode on carbon paper, and a Tokuyama A006 anion conductive membrane (active surface area of 5 cm^2^). Under such experimental conditions, in addition to hydrogen produced at the Pt/C cathode, they obtained a mixture of products at the anode; liquid chromatography and ^13^C NMR analyses indicated that glycerate, tartronate, glycolate, oxalate, formate, and carbonate were formed with glycerate and tartronate representing more than 70 % of the formed products.

**Figure 6:**
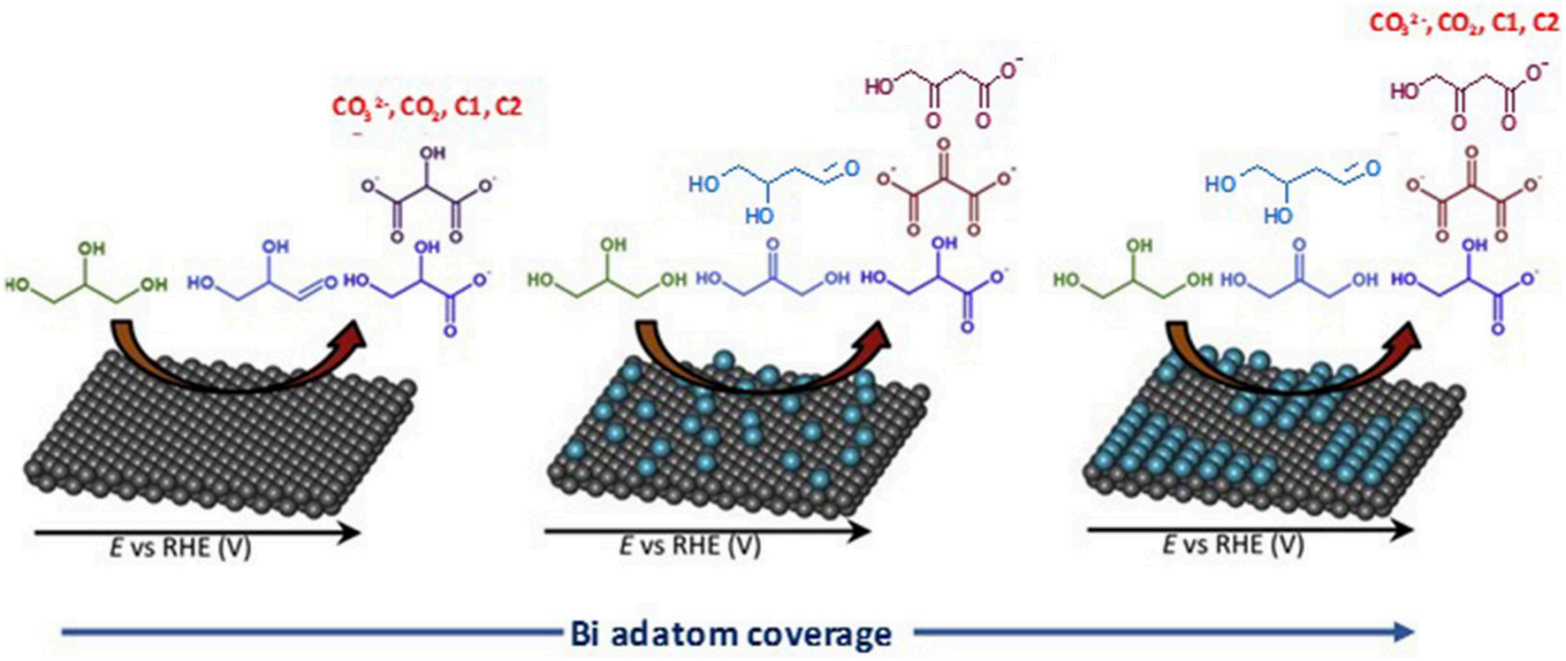
Influence of the coverage of a Pd surface by Bi adatoms on selectivity of glycerol oxidation reaction (Zalineeva et al., 2015).

Modification of palladium by p-group elements, such as indium (Serov et al., 2013), bismuth (Coutanceau et al., 2014; Zalineeva et al., 2014), and tin (Zalineeva et al., 2015), wad also studied for glycerol electrooxidation. Zalineeva et al. (2013), Zalineeva et al. (2015) studied the effect of the coverage of Pd-shaped nanoparticles by bismuth adatoms on the electrooxidation of glycerol. On pure Pd nanoparticles, they observed a higher electrocatalytic activity with nano-cubes (ca. 10 nm edges) presenting mainly extended (100) surface domains than with nano-octahedrons (ca. 8 nm tip to tip) presenting mainly extended (111) surface domains; spherical Pd nanoparticles (ca. 5 nm diameter) without preferential surface domain orientations led to the lowest activity. They also observed that the modification of the Pd surfaces by spontaneous adsorption of bismuth enhanced the activity for glycerol electrooxidation and that the coverage level of Pd by Bi adatoms did affect the reaction pathway of glycerol electrooxidation as a function of the electrode potential (Figure 6). Bi-coverages between 0.2 and 0.3 appeared to greatly limit the formation of CO_2_ and carbonate, i.e., the breaking of the C-C bond, and to increase the selectivity toward C3 compounds, particularly dihydroxyacetone and hydroxypyruvate.

**Figure 7:**
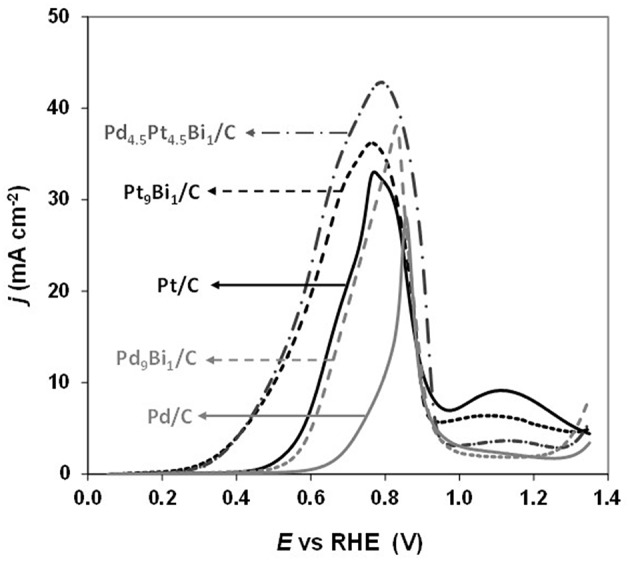
Polarization curves recorded for the oxidation of 0.1 M glycerol in N_2_-purged 1.0 M NaOH electrolyte on Pt/C, Pd/C, Pd_9_Bi_1_/C, Pt_9_Bi_1_/C and Pd_4.5_Pt_4.5_Bi_1_/C catalysts synthesized by the Water-in-Oil microemulsion method (scan rate = 10 mVs^−1^, *T* = 20°C) (Simões et al., 2011, 2012).

Simões et al. (2011, 2012) observed that a Pd_9_Bi_1_ (atomic ratio) catalyst and a Pt catalyst dispersed on a carbon powder (Vulcan XC 72) at 40 wt%, both with nanoparticles mean size of ca. 5 nm and prepared by the water-in-oil microemulsion method, led to the same activity toward glycerol electrooxidation from the onset potential of 0.4V vs. RHE (against 0.6 V vs. RHE for Pd/C, Figure 7). The same mechanism as for Pt/C was also observed on Pd_9_Bi_1_/C with the formation of glyceraldehyde and glycerate at potentials lower than 0.8 V vs. RHE and carboxylates at higher potentials (Figure 4D). Zalineeva et al. (2014) prepared self-supported Pd_x_Bi catalysts having a nanofoam structure by the sacrificial support method. *In situ* Fourier transform infrared spectroscopy highlighted the high selectivity as a function of the electrode potential: aldehyde and ketone at low potentials, hydroxypyruvate at moderate potentials, and CO_2_ at high potentials. The formation of hydroxypyruvate with high selectivity at moderate potentials (between 0.6 to 0.8 V vs. RHE), which was not observed with other Pd-based catalysts, demonstrated the importance of the catalyst structure/morphology on the selectivity. This unique catalytic behavior was explained in terms of confinement of reactants and intermediates in the catalyst pores acting as nanoreactors. These authors also synthesized self-supported Pd_1_Sn_x_ catalysts displaying the same nanofoam structure as Pd_1_Bi_x_ catalysts. The Pd_1_Sn_1_ material displayed the lowest glycerol oxidation onset potential (ca. 0.55 V vs. RHE) and led to the higher current density. According to *in situ* Fourier transform infrared spectroscopy, the formation of glyceraldehyde and glycerate did occur as soon as the Pd_1_Sn_1_ catalyst become active for the glycerol electrooxidation. No evidence of the C-C bond breaking leading to the formation of C1 compounds was observed at high potentials. Pd-Sn was then shown as being extremely selective toward the formation of C3 carboxylates from 0.55 V vs. RHE.

**Figure 8:**
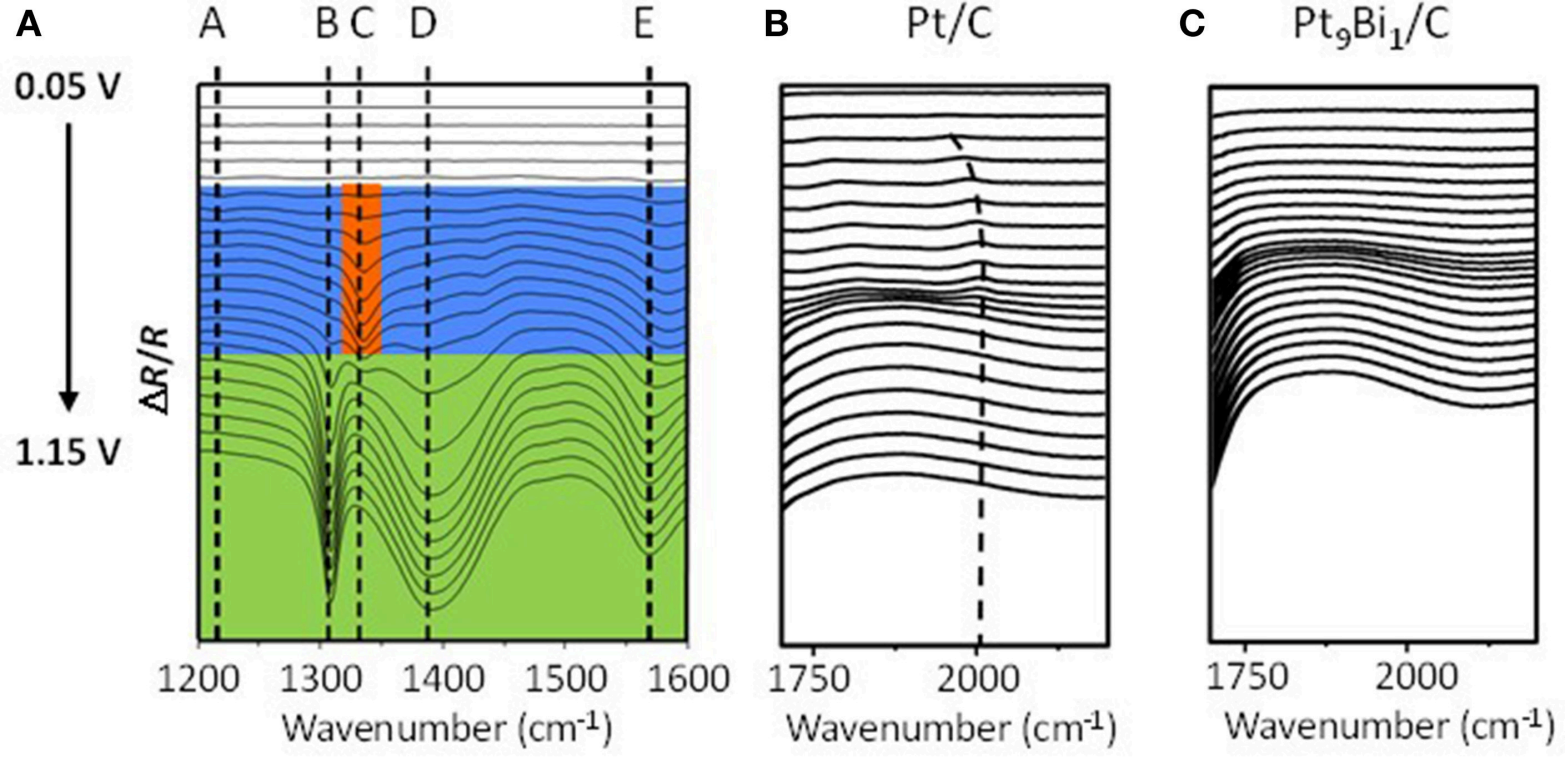
**(A)** Infrared spectra recorded in the 1,200–1,600 cm^−1^ range during 0.1 mol L^−1^ glycerol oxidation in 1.0 mol L^−1^ NaOH electrolyte from 0.05 V to 1.15 V vs. RHE on a Pt_9_Bi_1_/C catalysts prepared by the Water-in-Oil microemulsion method (*T* = 20°C, scan rate: 1 mV s^−1^, resolution 4 cm^−1^); **(B,C)** Infrared spectra recorded in the 1,700–2,300 cm^−1^ range during 0.1 mol L^−1^ glycerol oxidation in 1.0 mol L^−1^ NaOH electrolyte from 0.05 V to 1.15 V vs. RHE on **(B)** a Pt/C catalyst and **(C)** a Pt_0.9_Bi_0.1_/C catalyst (*T* = 20 °C, can rate: 1 mV s^−1^, resolution 4 cm^−1^) (Simões et al., 2011, 2012).

Simões et al. (2011, 2012) also studied the catalytic behavior of Pt and PtPd nanoparticles modified by bismuth clusters prepared by the water-in-oil microemulsion method. The addition of bismuth led to a dramatic decrease of the glycerol oxidation onset potential from 0.4 V vs. RHE on Pt/C to ca. 0.2 V vs. RHE on Pt_9_Bi_1_/C and Pt_4.5_Pd_4.5_Bi_1_/C catalysts, which made these catalysts among the most active ones at low electrode potentials. On *in situ* infrared spectra recorded for the electrooxidation of 0.1 M glycerol in 1.0 M NaOH electrolyte on Pt_9_Bi_1_/C (Figure 8A), a first absorption band appearing at ca. 1,225 cm^−1^ as soon as 0.2 V vs. RHE, i.e., as soon as the Pt_9_Bi_1_/C catalyst started to be active for glycerol oxidation, was assigned to the formation of glyceraldehyde. Another band at ca. 1335 cm^−1^ appeared from ca. 0.3 V to ca. 0.7 V which corresponded to the formation of dihydroxyacetone. For higher potentials, the absorption peaks located at ca. 1310 cm^−1^, 1385 cm^−1^, and 1570 cm^−1^ were assigned to the formation of glycerate and other carboxylate ions. The very high selectivity at low potentials of Pt_9_Bi_1_/C and Pt_4.5_Pd_4.5_Bi_1_/C toward aldehyde and ketones was explained in terms of absence of water activation on these catalysts for potential lower than 0.6 V vs. RHE, and further the non-occurrence of the bifunctional mechanism. At last, *in situ* infrared measurements indicated that the presence of Bi led to avoid the formation of adsorbed CO species on the Pt surface since the absorption bands between ca. 1,950 and 2,000 cm^−1^ assigned to adsorbed CO species visible on Pt/C (Figure 8B) could not be detected in spectra recorded on Pt_9_Bi_1_/C (Figure 8C) for the glycerol oxidation. This meant that the presence of bismuth made Pt less efficient for the C-C bond breaking and further that Pt_0.9_Bi_0.1_/C led to higher selectivity into C3 compounds.

**Figure 9:**
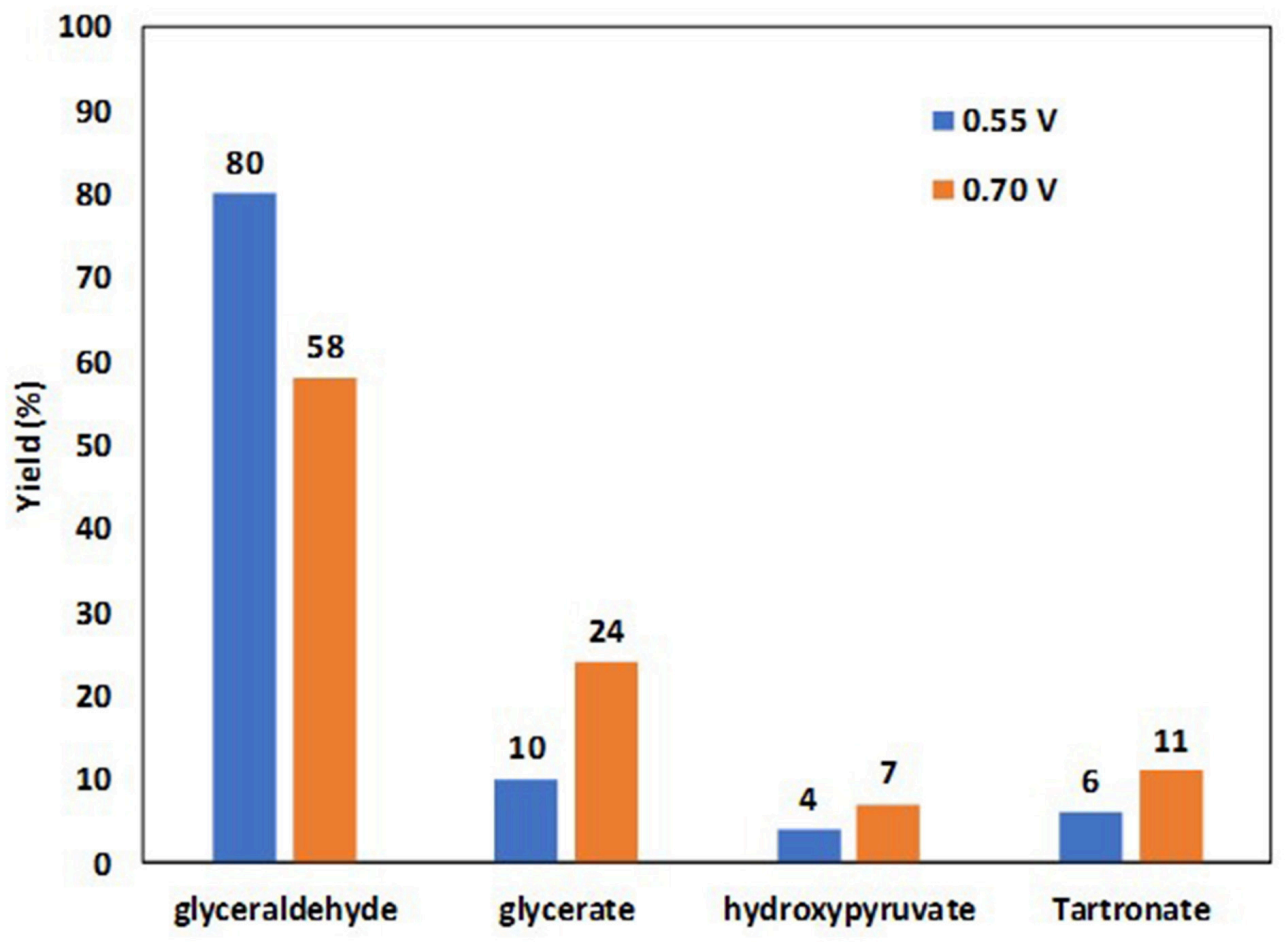
Yields in glyceraldehyde, glycerate, hydroxypyruvate and tartronate obtained by HPLC analyses of the reaction products after 4 h of electrolysis at cell voltages of 0.55 and 0.70 V (2.0 M glycerol in 0.5 M NaOH electrolyte, *T* = 20°C) at a Pt_9_Bi_1_/C anode (Cobos-Gonzalez et al., 2016).

Cobos-Gonzalez et al. (2016) confirmed these *in situ* infrared spectroscopy results by HPLC analysis of reaction products obtained after chroamperometry measurements in an electrolysis cell fitted with a 5 cm^2^ Pt/C cathode for the hydrogen evolution reaction, a Pt_0.9_Bi_0.1_/C anode for the glycerol oxidation reaction (metal loading of the electrodes 1.6 mg cm^−2^ and Nafion loading ca. 0.8 mg cm^−2^) placed on each side of a simple blotting paper as separator and mechanically pressed in the cell. The measurements were performed at 20°C in a 2 M glycerol and 0.5 M NaOH aqueous solution at cell voltages of 0.55 V and 0.7 V for 240 min. Figure 9 displays the yields obtained in the different compounds. At a cell voltage of 0.55 V (corresponding to an anode potential of ca. 0.6 V vs. RHE, considering that hydrogen evolution reaction at Pt/C is a rapid reaction and that the cathode potential remained close to −0.05 V vs. RHE), a very high selectivity into the formation of glyceraldehyde was obtained (yield of ca. 80 %), whereas at 0.70 V (corresponding to an anode potential of ca. 0.75 V vs. RHE) the selectivity toward carboxylate increased at the expense of that of glyceraldehyde (yield of ca. 58 %).

Recently, Zhou et al. (2013) studied the selective electrooxidation of glycerol on Pt_x_Au_y_@Ag catalysts. Pt_4_Au_6_@Ag displayed the highest activity in both acidic and alkaline media, the higher current densities being reached in alkaline medium. The product distribution from glycerol oxidation at 0.5, 0.7, 0.9, 1.1, and 1.3 V were analyzed by HPLC, and it was found that the catalysts led to a mixture of nine C1 to C3 acids together with glyceraldehyde and dihydroxyacetone. However, the Pt_4_Au_6_@Ag catalyst allowed obtaining the largest DHA selectivity of 77 % at 1.1 V.

## Conclusion

A comprehensive short overview of the electrooxidation of glycerol with the aim at producing value-added chemicals is presented in this contribution. The objective was to highlight some trends which could allow increasing both the glycerol conversion rate and the selectivity toward given compounds or chemical functions.

In acidic media, platinum is unavoidable for this electrocatalytic reaction. On pure platinum, the onset glycerol oxidation potential achieved was not lower than 0.4 V vs. RHE and a relatively low selectivity (mixture of compounds) was obtained. The modification of platinum by p-group elements allowed decreasing the onset potential and obtaining high selectivity toward dihydroxyacetone (with Bi and Sb) or glyceraldehyde (with Pb, Sn, and In) over a small potential range before a mixture of C3, C2, and C1 carboxylic acids started to be formed.

In alkaline media, non-noble metals, particularly nickel-based catalysts, become stable and active for the electrooxidation of glycerol. But the reaction occurs at very high potentials (higher than 1.1 V vs. RHE) and with low selectivity into C3 compounds, leading to a mixture of C3, C2, and C1 molecules. Gold also become active in alkaline media, as active as palladium, leading both to an onset potential of ca. 0.6 V vs. RHE. However, gold is less prone to poisoning by adsorbed species than Pd, which make it more selective toward C3 carboxylates and hydroxypyruvate. Pt remains still the most active materials in alkaline media, with an onset potential of ca. 0.4 V vs. RHE but leads to the same reaction pathway as Pd involving the formation of glyceraldehyde and dihydroxyacetone as first intermediates and glycerate/tartronate as secondary reaction products, as well as C-C bond breaking with adsorbed CO species and C1 to C3 compounds.

The modification of Pd/C nanocatalysts by d-group elements decreases the onset potential of glycerol oxidation, but does not change the selectivity, which remains the same as with pure Pd/C or pure Pt/C. The modification of Pd catalysts by p-group elements, such as bismuth and Sn, also leads to lower onset potentials of glycerol oxidation and higher activity and to changes in selectivity. The first important observation is that the presence of bismuth and tin on Pd surfaces leads to avoid the dissociative adsorption of glycerol (C-C bond breaking) and the formation of adsorbed CO species. Therefore, the selectivity toward C3 carboxylates is enhanced. It is worst also to note that the structure and morphology of the catalysts play a role for the selectivity the selectivity: glycerate and tartronate with Pd/C and hydroxypyruvate with Pd nanofoam.

The modification of Pt/C by bismuth leads to decrease the onset potential of glycerol oxidation to very low values (ca. 0.2 V vs. RHE). As for Pd, it leads to avoid the C-C bond breaking over a large potential range. The very wide potential range of activity provides different potential regions where different selectivities are obtained. Between 0.2 V vs. RHE and 0. 55 V vs. RHE, a Pt_9_Bi_1_/C catalyst display very high selectivity toward glyceraldehyde and dihydroxyacetone, whereas for potential between 0. 6 and 1.0 V vs. RHE, this catalyst presents very high selectivity into C3 carboxylates, particularly glycerate.

From these results, it seems that a lower onset potential of glycerol oxidation, a wider potential range of activity of a catalyst and a lower ability of the catalytic surface to adsorb dissociatively glycerol are the key parameters for achieving the best selectivity toward a unique desired compound at the best conversion rate.

## Author Contributions

All authors listed have made a substantial, direct and intellectual contribution to the work, and approved it for publication.

### Conflict of Interest Statement

The authors declare that the research was conducted in the absence of any commercial or financial relationships that could be construed as a potential conflict of interest.
